# Supervising Ethically: Applied Research Ethics Knowledge Among Supervisors of Medical Student Research Projects

**DOI:** 10.1007/s40670-026-02876-4

**Published:** 2026-09-09

**Authors:** Joanne Hart, Richmond Jeremy, Rajneesh Kaur

**Affiliations:** 1 School of Medicine, Faculty of Medicine and Health, University of Sydney, Sydney, Australia; 2 Central Clinical School, Faculty of Medicine and Health, University of Sydney, Sydney, Australia

**Keywords:** Research supervision, Research ethics, Medical education, Applied ethics, Research governance, Ethics education

## Abstract

Research training is a core component of contemporary medical education, and supervisors play a central role in ensuring that student research involving human participants is conducted ethically. Despite this, limited evidence exists on how supervisors apply ethical research principles in practice. This cross-sectional study examined research ethics experience and applied ethics knowledge among academic staff, clinicians, and affiliated researchers supervising medical student projects in a large Australian Doctor of Medicine (MD) Program (2023–2024). An online survey collected demographic data, research experience, ethics training, and responses to scenario-based questions addressing consent, recruitment, privacy, risk management, and incidental findings. Seventy-seven supervisors completed the survey (response rate 24%), and sixty-four completed the ethics knowledge section (response rate 20%). Participants reported substantial research experience and high engagement with national ethics frameworks. Supervisors performed strongly on some items, including consent for use of medical records and confidentiality in interview-based research, while performance was more variable for clinic-based recruitment, participant selection, guiding ethical principles and incidental findings. Subgroup analyses showed no consistent pattern of association between supervisor characteristics and performance, although several item-level associations were observed. These findings suggest that familiarity with ethics governance frameworks does not consistently translate into applied ethical judgement. For medical educators and faculty developers, these findings highlight the need for supervisory development programs that go beyond policy-based training. Strengthening applied ethical reasoning requires educational approaches that prioritise reflective, scenario-based engagement with ethical decision-making.

**Supplementary Information:**

The online version contains supplementary material available at https://doi.org/10.1007/s40670-026-02876-4.

## Introduction

Medical student research projects are now a standard feature of graduate-entry medical curricula internationally, and they frequently involve human participants, clinical data, and vulnerable populations [1, 2]. Participation in research during medical training is associated with ongoing engagement in academic medicine and the development of clinician-scientists [3–5], and supervised student research projects are embedded within medical curricula across a range of disciplines and settings. In this context, ethical conduct is central to both participant protection and the professional formation of future clinician-researchers. Research supervisors have an important responsibility for the ethical conduct of student projects: they guide methodological decisions, support students to interpret research governance requirements, and model how ethical principles are enacted in practice [6, 7]. Supervisory practice may therefore influence not only the conduct of individual projects but also students’ developing understanding of research integrity and professional responsibility [7, 8].

Research ethics education for medical students can include formal curriculum, online human-participant protection or responsible-conduct modules, and project-specific learning embedded within supervised research [9, 10]. Formal instruction provides an important foundation in principles and regulatory requirements, but supervised research also exposes students to the practical interpretation of those requirements in real decisions. Supervisors therefore contribute to ethics education through both explicit discussion and role modelling [7, 8]. Deliberate discussion may be particularly important when students encounter context-dependent issues such as voluntariness in clinical recruitment, confidentiality, participant selection and incidental findings.

Research ethics governance frameworks provide the regulatory foundation for supervisory practice. In Australia, the National Statement on Ethical Conduct in Human Research [11] and the Australian Code for the Responsible Conduct of Research [6] articulate principles and procedural requirements grounded in respect for persons, beneficence, and justice [12]. Supervisors are expected to know and apply these frameworks. Yet applying principles within specific contexts, such as managing recruitment in a clinical setting, responding to incidental findings, or selecting participants fairly, requires more than regulatory knowledge. It requires what has been described as practical wisdom, or *phronesis*: the capacity to perceive the ethically salient features of a particular situation and respond appropriately [13]. This distinction between procedural governance knowledge and contextual ethical judgement is central to understanding what competent research supervision demands.

Empirical studies have examined research ethics knowledge across a range of healthcare education populations. Weston et al. [9] surveyed supervisors and students at an Australian medical school using a scenario-based instrument, finding that supervisors generally outperformed students but that significant knowledge gaps remained, particularly in areas of recruitment and consent. Munoli et al. [14] examined knowledge, attitudes and practices among medical faculty at a teaching hospital in India and reported limited familiarity with ethical principles governing recruitment and participant selection. Tarboush et al. [15] studied health sciences students and faculty in Jordan and identified comparable deficiencies in applied ethics knowledge, particularly in domains relating to voluntariness and informed consent. Collectively, these studies identify variability in applied ethics knowledge, but their populations have been mixed, and the focus has not been specifically on research supervisors in their educative role. Research supervisors, defined here by their dual responsibility as practising researchers and educators who influence students’ ethical development, have received limited focused attention. It is not known whether familiarity with governance frameworks translates into the contextual ethical reasoning that supervision demands. This gap is particularly salient in distributed medical education settings, where supervisors include academics, clinicians and affiliated researchers with varied exposure to formal training and institutional oversight [16].

This is important for medical education because if variability in supervisory ethical reasoning cannot be explained by experience, qualifications, or prior training, then standard compliance-based approaches to faculty development are insufficient. Understanding where applied ethics reasoning breaks down, and why, is a prerequisite for designing supervisory development programs that strengthen ethical practice rather than simply reinforcing regulatory awareness.

This study examined the human research ethics experience and applied ethics knowledge of supervisors of medical student research projects within a large Australian medical program. Specifically, it addressed three research questions: (1) What is the level of applied research ethics knowledge among supervisors, as assessed through scenario-based questions? (2) In which ethical domains do supervisors perform strongly or variably? (3) Are individual supervisor characteristics, including staff category, qualifications, ethics training, and human research ethics committee experience, associated with applied ethics performance?

## Methods

### Study Design

This study used a cross-sectional survey design to examine applied research ethics knowledge and related experience among supervisors of medical student research projects. An online self-report survey was administered to all eligible supervisors in a single large Australian graduate-entry medical program during 2023 and 2024. The survey combined closed questions assessing participant characteristics, research experience, and ethics training with scenario-based multiple-choice questions measuring applied ethics knowledge across five domains: consent, recruitment, risk management, privacy and confidentiality, and management of incidental findings.

### Educational Context

The University of Sydney’s four-year graduate-entry Doctor of Medicine (MD) Program enrols around 300 students per year from diverse academic and professional backgrounds. An integral and compulsory component of the curriculum is the research project (MD Project), run in a 14-week full-time block in Year 3 of the program. The MD Project requires students to formulate research questions and hypotheses, conduct literature reviews, acquire appropriate methodological skills, collect and/or analyse data, and prepare a scientific report. Projects span a wide range of disciplines, and include clinical research, biomedical science, epidemiology, public health, medical education, bioinformatics, information technology, policy, law, and ethics. They may involve prospective or retrospective designs, laboratory or clinical work, or secondary analyses of curated datasets or published literature. Projects are undertaken across ten metropolitan and rural clinical schools and multiple affiliated research institutes.

Students receive structured preparation in human research ethics through a two-hour lecture on human research ethics and approval processes, a two-hour workshop on ethical issues arising in MD Projects, and an assessment requiring appraisal of the ethical issues relevant to their own project. Students named on a project approved by the institutional Human Research Ethics Committee (HREC) must also complete a 45-minute online ethics training module, which is also available to and recommended for all students.

Supervisors may oversee up to four individual student projects concurrently, reflecting the scale and complexity of the program. Students are supervised individually or in small groups by academic staff and University affiliates, including clinicians, biomedical scientists, and public health or medical education researchers. Most supervisors are not directly employed by the School of Medicine and are not specifically remunerated for their supervisory roles. They are not required to hold doctoral qualifications or formal training in research supervision, and no dedicated faculty funding is provided for supervision. Within this program, supervisors typically provide project resources, methodological expertise and academic guidance, often alongside substantial clinical, administrative and research workloads [16]. Many are based in public hospitals and supervise medical student research projects in addition to their existing professional responsibilities.

### Participants

Eligible participants were academic staff, conjoint clinical staff, and university affiliates who supervised medical student projects in 2023 or 2024 (*n* = 319).

### Survey Instrument

The survey used items from previously published instruments used to assess research ethics knowledge among clinical researchers, supervisors and medical students [9, 17], with minor modifications to scenario wording to reflect current Australian guidance and the supervisory context. Scenarios were reviewed for relevance to contemporary supervisory practice and alignment with current national guidance [6, 11]. Correct responses were anchored to the National Statement and Australian Code. Two members of the research team reviewed the survey for clarity. Section 1 defined ethics training as participation in recognised programs such as university modules, Good Clinical Practice training or institutional ethics induction, and human research ethics committee (HREC, broadly equivalent to an Institutional Review Board (IRB)) review experience as having acted as a named reviewer for at least one ethics application.

**Section 1** comprised 20 questions about participant characteristics, research experience, ethics training, engagement with the National Statement on Ethical Conduct in Human Research [11] and the Australian Code for the Responsible Conduct of Research [6], and HREC experience.

**Section 2** comprised scenario-based multiple-choice questions addressing the following ethical issues. The ethical principle or concern shown in parentheses identifies the primary issue represented by each item to aid interpretation; individual scenarios may engage more than one ethical principle:

- Use of patient information (respect, privacy and confidentiality).
- Consent requirements (respect for persons and informed consent).
- Participant information requirements (respect for persons and informed consent).
- Recruitment practices (respect, voluntariness and minimisation of undue influence).
- Ethical principles guiding HREC review (recognition and application of National Statement principles).
- Fair participant selection (justice).
- Risk–benefit management (beneficence, non-maleficence and risk minimisation).
- Privacy and confidentiality (respect and privacy).
- Researcher responsibilities regarding incidental findings (beneficence, participant welfare and researcher role boundaries).

Scenarios reflected common supervisory contexts in medical student research and were aligned with national guidance documents [6, 11].

**Section 3** included a free text field inviting participants to indicate the type of information, education or training about human research ethics that they would find useful.

The survey tool is provided as a supplementary file.

### Data Collection

Supervisors were invited via email to complete an online survey. The survey remained open for 4 weeks, and a reminder email was sent 2 weeks into the open period. Participation was voluntary; consent was implied through survey submission. Data were collected using Qualtrics (Qualtrics, Provo, UT, USA).

### Data Analysis

Descriptive statistics summarised participant characteristics and scenario responses. Categorical comparisons were analysed using Pearson chi-square or Fisher’s exact tests where appropriate. Subgroup analyses examined associations with staff category, medical degree, doctoral qualification, prior ethics training and HREC reviewer experience. Staff category was analysed as university academic/affiliate, conjoint medical/health professional, and university non-academic staff, consistent with Table 1. Statistical significance was set at *p* < 0.05. The subgroup analyses were exploratory, and the study was not powered a priori for these comparisons; therefore, no adjustment for multiple comparisons was applied and findings were interpreted cautiously. Analyses were performed using IBM SPSS Statistics, version 29 (IBM Corp., Armonk, NY, USA).

**Table 1:** Participant characteristics

Characteristic	*n *(%) Total *n* =64
Gender	
Male	27 (42)
Female	37 (58)
Age (Years)	
20–29	1 (2)
30–39	18 (28)
40–49	14 (22)
50–59	24 (38)
60+	7 (10)
Qualification*	
Medical degree	36 (56)
Doctorate (PhD)	50 (78)
Post-graduate Fellowship	31 (48)
Staff category	
University Academic staff or affiliates	44 (69)
Conjoint medical/health professional staff	17 (27)
University non-academic staff	3 (4)

*Many held multiple qualifications so will not total to 100%

## Results

### Survey Participants and Responses

Of 319 invited supervisors, 77 completed the survey (response rate 24%). Of these, 64 completed the ethics knowledge section (response rate 20%), reflecting section-level non-response.

### Participant Characteristics

Participant characteristics are summarised in Table 1. The sample was predominantly female (58%) and included participants across early to later career stages, with the largest proportion (88%) in mid-career age groups. Most participants reported high levels of academic and professional qualification, with many holding medical degrees, doctoral qualifications, and/or post-graduate fellowships. The group was largely composed of university academic staff or affiliates, alongside conjoint medical staff and a smaller number of university non-academic staff.

### Participant Experience with Human Research

Overall, 60 participants (94%) reported prior experience conducting human research. Most indicated long-standing involvement in human research, with 73% reporting more than five years of experience (Table 2). Human research formed a central component of participants’ scholarly activity, with the majority reporting that most or all their research was conducted within health and medical contexts.

**Table 2:** Experience with research involving humans

Human research experience	*n* (%) Total *n*=60
Length of involvement in human research	
<1 year	1 (2)
1–2 years	7 (12)
2–5 years	8 (13)
>5 years	44 (73)
Proportion of human research in health and medicine	
None	1 (2)
Some	2 (3)
Most (>50%)	14 (23)
All (100%)	43 (72)
Number of human research projects involved in	
<5	6 (10)
5–10	17 (28)
>10	37 (62)
Number of ethics applications	
<5	13 (22)
5–10	21 (35)
>10	25 (41)
Not answered	1 (2)
Number of publications from human research	
<5	9 (15)
5–10	11 (18)
>10	40 (67)

Participants also reported extensive experience in human research projects and outputs. 62% had been involved in more than ten human research projects, and 67% reported more than ten publications arising from human research. Overall, the sample reflects a group with substantial experience in conducting and disseminating human research.

### Participant Training in Human Research Ethics

Participants reported high levels of engagement with formal research ethics guidance and training (Table 3). Most indicated that they had read all or part of the National Statement on Ethical Conduct in Human Research [11] and the Australian Code for the Responsible Conduct of Research [6], and the majority had attended training related to the ethical conduct of human research. A smaller proportion reported experience acting as reviewers for HREC applications, indicating exposure to the ethics review process from a reviewer perspective. Four participants did not respond to the HREC reviewer-experience item; missing responses are shown separately in Table 3.

**Table 3:** Research ethics training and experience

Training or experience	Response *n* (%) Total *n*=64
Read all or part of the National Statement on Ethical Conduct in Human Research	Yes: 55 (86); No: 9 (14)
Read all or part of the Australian Code for the Responsible Conduct of Research	Yes: 53 (83); No: 11 (17)
Attended training in the ethical conduct of human research	Yes: 55 (86); No: 9 (14)
Acted as reviewer for HREC applications	Yes: 19 (30); No: 41 (64); Missing: 4 (6)

Participants reported completing a range of research ethics training, including university-based courses, higher degree workshops, Good Clinical Practice (GCP) training, and mandatory institutional modules. Training was delivered through both online and face-to-face formats and was often required by universities or ethics committees. Some participants also reported undertaking external or internationally recognised programs, with periodic refresher training undertaken.

### Scenario-based Ethics Knowledge

Ethics knowledge was assessed using scenario-based questions addressing consent and participant information, recruitment and ethical principles, and management of risk, privacy, and incidental findings. Findings are synthesised under three domains:

#### Consent and Participant Information

Consent and Participant Information

Performance across the consent and participant-information items was mixed (Table 4). Most respondents recognised the need for consent when medical records were used, but fewer selected the complete set of circumstances requiring written consent (38%). Correct identification of all circumstances requiring a written Participant Information Statement was more common (63%).

**Table 4:** Requirements for written consent and participant information

Scenario	Written Consent Form required *n* (%) Total *n*=64	Participant Information Statement required *n* (%) Total *n*=64
a. when a research participant is invited to complete an anonymous survey	25 (39)	48 (75)
b. when a research participant is asked for access to his/her medical records	52 (81)	50 (78)
c. when a research participant is asked to be part of a drug trial	52 (81)	53 (83)
d. when a research participant is asked for health information that may identify them to others	52 (81)	52 (81)
e. when a research participant is from a vulnerable group	45 (70)	47 (73)
Correct answer	24 (38) (all except a)	40 (63) (all a-e)

*Correct responses reflect principles of informed consent, respect for persons, and appropriate provision of participant information*

#### Recruitment and Ethical Principles

Responses to the scenarios addressing recruitment and ethical principles showed variable application of respect, voluntariness and justice (Table 5). Fewer than half identified respect as the principle most directly engaged in the low-risk scenario, and only 20% selected passive, patient-initiated contact as the preferred clinic-based recruitment approach. Most instead selected a receptionist-mediated approach, indicating uncertainty about how voluntariness and potential influence operate in a clinical setting.

**Table 5:** Responses to recruitment and ethical principles scenarios

Scenario question	Scenario	*n* (%) Total *n*=64
Ethical principle prioritised by HREC in low-risk recruitment	Respect *(correct)* Beneficence Justice Research merit Don’t know	28 (44) 21 (33) 11 (17) 2 (3) 2 (3)
Preferred recruitment strategy in clinic-based research	Passive notice inviting patient-initiated contact *(correct)* Receptionist provides information and asks if contact details can be shared Receptionist informs patients that the researcher is available Don’t know	13 (20) 47 (73) 3 (5) 1 (1)
Fair participant selection in emergency department family interviews	All family members attending on a selected day *(correct)* Any family member who volunteers Don’t know	24 (38) 36 (56) 4 (6)

*Correct responses reflect ethical principles of respect for persons, voluntary participation, minimisation of coercion in recruitment, and justice in participant selection*

In the separate scenario addressing fair participant selection for interviews with family members in an emergency department setting, 38% identified inclusion of all eligible family members attending on a defined day as the fairest selection strategy, whereas 56% selected volunteer sampling. This item concerns the justice of participant selection rather than the mechanics of recruitment; accordingly, it is interpreted separately from the clinic-based recruitment scenario.

#### Risk, Privacy, and Incidental Findings

Responses to scenarios examining risk management, privacy, and responsibilities arising from incidental findings are summarised in Table 6.

**Table 6:** Responses to scenarios addressing risk management, confidentiality, and incidental findings

Scenario question	Scenario	*n* (%) Total *n*=64
Managing risk in mobility research with older patients	Individualised mobility measures *(correct)* Maintain effort at level tolerated by least mobile participant Escalating weekly exercise targets Advise all participants of a validated mobility scale they should reach Don’t know	43 (67) 7 (11) 4 (6) 3 (5) 7 (11)
Confidentiality in interview research with health professionals	Remove identifying information about participants and third parties *(correct)* Use pseudonyms only Retain identifiers but not report them Don’t know	53 (83) 7 (11) 3 (5) 1 (2)
Researcher responsibilities when depressive symptoms identified *(Multiple answers)*	Include information about symptoms of depression *(correct)* Include information on where to seek help *(correct)* Both correct answers Inform the participant’s GP Refer participant to counselling or mental health service Inform practice staff Approach participant about symptoms	37 (58) 58 (91) 17 (27) 6 (9) 3 (5) 23 (36) 17 (27)

*Correct responses reflect principles of risk minimisation, confidentiality, and appropriate management of incidental findings in research*

In the mobility-intervention scenario, approximately two-thirds identified an individualised approach as the most appropriate means of balancing risk and benefit (Table 6), indicating comparatively strong performance on this item. Performance was also strong on the confidentiality scenario: 83% identified removal of identifying information relating to both participants and third parties as the appropriate approach (Table 6). Responses were more variable for the incidental-findings scenario. Although most recognised that participant information should include advice about where help could be sought, only 27% selected both responses designated as appropriate; other responses suggested uncertainty about the boundary between researchers’ responsibilities and direct clinical intervention (Table 6).

### Subgroup Analyses

Subgroup analyses identified no consistent pattern of association between supervisor characteristics and scenario-based ethics performance (Supplementary Table S1). PhD qualification and HREC reviewer experience were not significantly associated with performance on any of the nine items. Participants with a medical degree were substantially less likely than those without a medical degree to identify the preferred clinic-based recruitment strategy (1/36 [2.8%] vs. 12/28 [42.9%], *p* < 0.001) but were more likely to identify the appropriate privacy and confidentiality protections in interview-based research (34/36 [94.4%] vs. 19/28 [67.9%], *p* = 0.007). Two additional nominal associations were observed: performance on the privacy/confidentiality item differed by staff category (*p* = 0.031), with correct responses from 34/44 (77.3%) university academic/affiliate staff, 17/17 (100%) conjoint medical/health professional staff and 2/3 (66.7%) university non-academic staff; and supervisors reporting prior ethics training were more likely to answer the risk-management item correctly than those without such training (40/55 [72.7%] vs. 3/9 [33.3%], *p* = 0.049). Given the exploratory nature, modest subgroup sizes and number of comparisons, these item-level findings should be interpreted cautiously.

### Further Training Needs

Participants were invited to suggest useful ethics information, education or training. Thirty-nine participants provided a substantive response to this free-text item; the remaining participants either did not respond or indicated that they did not require further training. Among those providing suggestions, the main training needs and preferences were:

1. Understanding Ethics Procedures: Researchers wanted to comprehend current ethics approval criteria, local Human Research Ethics Committee rules, and the process of ethics review.
2. Up-to-Date Information: Access to updated information on research ethics requirements, national protocols, guidelines, and changes to regulations are essential, preferably delivered online.
3. Practical Resources: Researchers seek resources and guidance for ethics application preparation, both in online and on-demand formats.
4. Scenario-Based Learning: A desire for interactive, scenario-based learning, preferably in small groups, to discuss common ethical dilemmas.
5. Accessible Training Formats: Researchers appreciate various training formats, including short courses, online sessions, and self-directed options.

Among supervisors who expressed a training preference, needs spanned both procedural support and opportunities to work through practical ethical problems. Requests for up-to-date guidance, local HREC information and assistance with applications emphasised the continuing value of accessible governance resources, while some respondents also preferred interactive scenario-based discussion. The latter preference is consistent with literature supporting active and problem-oriented approaches to ethics instruction [18].

## Discussion

This study found that substantial research experience and familiarity with formal ethics frameworks did not necessarily translate into consistently strong applied ethical judgement among supervisors of medical student research projects. Performance was strongest where scenarios aligned closely with established procedural expectations but became more variable when supervisors were required to interpret ethical principles within context-dependent situations. Supervisor characteristics did not show a consistent relationship with performance, suggesting that applied ethical judgement is not readily explained by qualifications, prior training, or experience alone.

### Interacting Components of Supervisory Ethics Competence

**Fig. 1:**
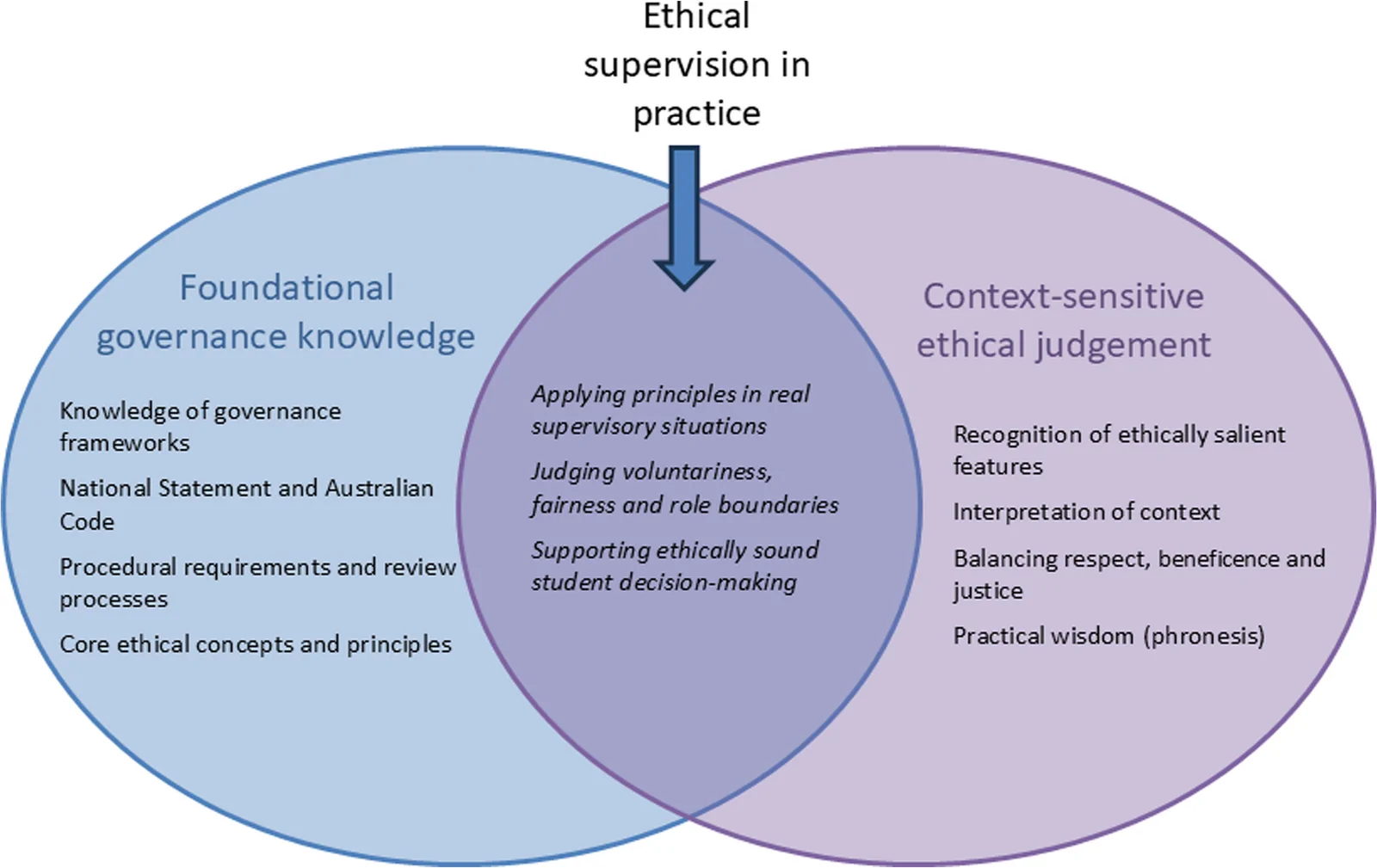
Interacting components of supervisory ethics competence. Procedural knowledge comprises familiarity with research governance frameworks and requirements, while applied ethical judgement involves interpreting and applying ethical principles in context. These components are complementary rather than mutually exclusive, and individual ethical scenarios may draw on both

Taken together, the findings are consistent with a distinction between knowing governance requirements and exercising context-sensitive ethical judgement. Figure 1 presents these as interacting components of supervisory ethics competence, rather than as separate categories of ethical knowledge. Individual domains such as consent, recruitment, risk management, privacy and incidental findings may draw on both procedural knowledge and contextual reasoning to differing degrees. In the clinic-based recruitment item, for example, recognising a formally permissible process is not sufficient without also considering voluntariness, power relationships and the influence of clinical staff. Responses to the incidental-findings scenario similarly required participants to distinguish participant welfare obligations from clinical responsibilities.

### Professional Context and Ethical Judgement

The recruitment finding among medically qualified supervisors may reflect the way ethical judgement is shaped by professional context. In clinical environments, staff-mediated approaches to patient contact are routine and may therefore appear appropriate or efficient, even when research recruitment requires additional attention to voluntariness, perceived obligation and power relationships. This is consistent with the broader literature showing that ethical decision-making is influenced not only by knowledge of formal requirements but also by professional norms and situational context [19–22]. The finding should nevertheless be interpreted cautiously. Medical qualification was not associated with consistently poorer performance across the scenarios, and the study did not explore participants’ reasoning for their choices. It is therefore better understood as a hypothesis-generating indication that familiar clinical practices may shape how recruitment ethics are interpreted, rather than as evidence of a deficit associated with medical training.

### Practical Wisdom in Research Ethics

This distinction aligns with broader conceptualisations of ethics as involving both principled guidance and contextual judgement. Governance frameworks such as the National Statement and the Australian Code are grounded in principlism [12], providing structured normative guidance. However, their application requires interpretation within specific contexts. This form of reasoning has been described as practical wisdom, or *phronesis* [13], reflecting the capacity to discern and respond to morally relevant features of particular situations. Consistent with prior research, the findings suggest that procedural knowledge alone may be insufficient for developing this form of judgement [9, 14, 15].

### Implications for Ethics Education

The findings also resonate with research on ethics education, which suggests that instruction focused primarily on rules and compliance may improve knowledge without necessarily producing equivalent gains in ethical decision-making [19, 23]. Ethical judgement is shaped not only by knowledge but also by reflection, experience and professional context. Supervisors are influential in this process because research integrity is communicated through both explicit guidance and day-to-day supervisory practice and role modelling [3, 7, 8]. Variability in supervisory reasoning may therefore influence the ethical norms and decision-making approaches encountered by students during their projects.

### Beyond Compliance-based Approaches

At an organisational level, these findings highlight the limits of compliance-based approaches to research ethics [23]. While governance systems are essential for standardisation and oversight, they do not necessarily foster shared ethical understanding or context-sensitive judgement. When ethics is framed primarily as a procedural requirement, engagement may shift toward regulatory compliance rather than reflective deliberation [20–22]. Embedding opportunities for discussion of real-world ethical dilemmas within supervisory practice may therefore be important for strengthening applied reasoning.

### Implications for Supervisor Development

For medical student research supervision, these findings have practical implications. Student projects may involve vulnerable populations and complex clinical contexts [1, 16], placing supervisors in important roles as ethical guides and role models. Evidence from ethics instruction in the sciences supports active approaches that require learners to practise ethical decision-making rather than relying on rule transmission alone [19], and scenario-based or deliberative formats are therefore reasonable candidates for supervisor development [18]. However, the present study did not test an educational intervention and cannot establish that such training would improve supervisory practice. The absence of better performance among participants with HREC reviewer experience also indicates that exposure to ethics review should not be treated as equivalent to deliberately designed education involving facilitated reasoning, feedback and reflection. The effectiveness of such supervisor-development approaches should be evaluated prospectively.

### Limitations

This single-program study had a modest response rate, which may limit generalisability and introduce non-response bias, the direction of which cannot be determined. Subgroup analyses were exploratory, based on small numbers, and involved multiple comparisons without adjustment; these findings should therefore be interpreted as hypothesis-generating. The source instruments have not been formally validated, and scenario-based questions assess recognition of preferred responses rather than ethical behaviour in practice.

## Conclusion

Overall, supervisors in this study demonstrated substantial research experience and engagement with formal ethics frameworks but applied ethics performance varied across common supervisory scenarios. Performance was stronger in areas such as consent, confidentiality and risk management, and more variable in clinic-based recruitment, participant selection and incidental findings. Supervisor characteristics did not show a consistent relationship with performance. These findings support viewing governance knowledge and context-sensitive ethical judgement as complementary components of supervisory competence and suggest that supervisor development should combine clear procedural guidance with opportunities to practise ethical reasoning in realistic contexts. Whether scenario-based and reflective approaches improve supervisory decision-making remains an important question for future evaluation.

## Supplementary Information

Below is the link to the electronic supplementary material.

Supplementary File 1 (PDF 192 KB)

Supplementary File 4 (PDF 96.3 KB)

## Data Availability

The de-identified datasets used and/or analysed during the current study are available from the corresponding author on reasonable request.
