# Supplementary material for: Supervising Ethically: Applied Research Ethics Knowledge Among Supervisors of Medical Student Research Projects
Source: Med Sci Educ. 2026 Sep 9;36(5):2327–36. doi: 10.1007/s40670-026-02876-4 (PMC13723458; doi:10.1007/s40670-026-02876-4)
Supplement: Supplementary file 1 [file 40670_2026_2876_MOESM1_ESM.pdf]

Research Supervisor Awareness and Understanding of Human Research Ethics

The purpose of this survey is to gather information about your knowledge and experience regarding requirements for research involving humans. Academic and conjoint staff and research supervisors involved in the MD Project in the Sydney Medical Program, University of Sydney have been invited to participate.

The ethical aspects of this study have been approved by the Human Research Ethics Committee (HREC) of The University of Sydney **[2021/853]** according to the *National Statement on Ethical Conduct in Human Research (2007)*.

The full Participant Information Statement is here [link to Participant Information Statement]

You do NOT need to have previously conducted research to complete this survey. Information from the survey will be used to design education and training to better support students and staff for research and supervision. The survey should take about 15-20 minutes to complete.

If you decide to participate, submission of the completed online form will be taken as consent to participate in the study. You may withdraw at any time for any reason, prior to the final submission.

Your participation is greatly appreciated. *Thank you!!*

[i] **Survey Questions**

A: ABOUT YOU

Please indicate that you have read and understood the Participant Information Statement for this research. Yes/No

1. Please indicate your gender

Male | Female | Other | Prefer not to say

2. Please indicate your age range

20 to 29 years | 30 to 39 years | 40 to 49 years | 50 to 59 years | 60 years and over

3. Please indicate your current position (fill in as many answers as apply):

University academic staff
Conjoint medical staff
Conjoint health professional staff
University non-academic (professional or general) staff
Higher degree research student (e.g., Hons, Masters, PhD)
Other, please specify ……………………………………………………………………………………

4. What qualification(s) do you have? ***Please fill in all qualifications you possess:***

High school matriculation (e.g. HSC)
An undergraduate degree (non-medical)
An Honours degree (non-medical)
A Masters degree (research)
A Masters degree (coursework, no research)
A Medical degree e.g. MBBS, MD
A Doctorate (PhD)
Postgraduate Fellowship (eg FRACP, FRACS)
Other, specify ___________________________

5. With regard to your most recent qualification, in what discipline area did you qualify?

Basic Science
Clinical Discipline
Population Health
Other, specify __________________________

B. YOUR RESEARCH EXPERIENCE

6. Have you previously conducted any research on humans?

No **If No, please go to Question 17**
Yes

If YES, please answer the following questions.

7. About how long (full time equivalent) were you involved in this research?

< 1 year
1-2 Years
2-5 Years
>5 years

8. How much of your research involvement was in a health or medical-related area?

All
Most (50% or more)
Some (less than 50%)
None

9. Please describe the field(s) of research:

10. Altogether, approximately how many <u>human research projects</u> - projects involving human participants, human tissue samples or using information about people - have you been involved in (ever) at all levels (e.g. as a student, an investigator or research assistant) and on any subject?

NONE
<5
5-10
>10

11. Have you ever published or co-authored any papers from a human research project in a peer-reviewed journal?

No ☞ If No, please go to Question 13
Yes

12. If yes, how many peer-reviewed human research papers publications have you had published in peer-reviewed journals?

<5
5-10
11-50
>50

13. Have you ever completed or assisted in the completion of a Human Research Ethics Application?

No If No, please go to Question 15
Yes

14. If Yes, how many Human Research Ethics applications have you completed or assisted with?

<5
5-10
11-50
>50

15. Are you or have you ever been a member of a Human Research Ethics Committee (HREC)?

No
Yes

16. Are you or have you ever been a reviewer for a HREC?

No
Yes

ALL PARTICIPANTS TO COMPLETE THE FOLLOWING SECTION

17. Have you ever read (at least in part) the National Statement on Ethical Conduct in Human Research, published by the National Health and Medical Research Council (NHMRC), Australian Research Council (ARC), and Universities Australia (UA)(2007)?

No
Yes

18. Have you ever read (at least in part) the Australian Code for the Responsible Conduct of Research (ARC, UA, NHMRC)?

No
Yes

19. Have you ever attended any training in the ethical conduct of undertaking human research?

No
Yes

20. If yes, please describe the type and duration of research ethics training you have undertaken:

C. ETHICS REQUIREMENTS WHEN UNDERTAKING RESEARCH INVOLVING HUMANS

NOTE: questions 21-28 ask for your opinion on ethical matters arising in short scenarios. We are interested in your familiarity with and your opinions on typical research ethics issues.

21. When can a doctor use information from the medical records of his/her patients for a research publication? ***Please fill in ONE answer.***

A when the doctor decides that using the information would advance scientific knowledge
B when the patient has given specific consent for that research project
C when patients have been informed that their records might be used for research
D when seven years has passed after the last consultation with that doctor
E don't know

22. When is a signed consent form required? ***Please fill in AS MANY answers as required.***

A when a research participant is invited to complete an anonymous survey
B when a research participant is asked for access to his/her medical records
D when a research participant is asked to be part of a drug trial
C when a research participant is asked for health information that may identify them to others
E when a research participant is from a vulnerable group, such as a non-English speaking background
F don't know

23. When is a written participant information sheet required? ***Please fill in AS MANY answers as required.***

A when a research participant is invited to complete an anonymous survey
B when a participant is asked for access to his/her medical records
C when a participant is asked to be part of a drug trial
D when a research participant is asked for health information that may identify them to others
E when a research participant is from a vulnerable group, such as a non-English speaking background
F don't know

24. You are undertaking a research project and would like to find out how patients at a local health clinic prepare themselves for a consultation. For example, how much reading they do, whether they prepare questions to ask the doctor.

(a)Which ONE of the following principles of research ethics is a reviewing HREC **most likely to focus on?** ***Please fill in ONE answer.***

A Research merit – how well your project is designed
B Justice – the fairness of your recruitment methods
C Beneficence – whether the risks to patients are justified
D Respect – how patients will be approached and give their consent for an interview
E don't know

This research project is approved by the HREC. You plan to conduct interviews with patients at the local health clinic.

(b)Which ONE of the following methods of gaining consent is preferred to achieve your research aims and treat patients ethically? ***Please fill in ONE answer.***

A Placing a notice on the reception desk informing patients of the project and asking them to telephone you if they are interested
B Arranging for the receptionist to tell each patient that the project is being conducted and that you are available in an office in the clinic
C Sitting in the waiting room and approaching patients yourself to ask if they are willing to be interviewed
D Arranging for clinic staff to give you the contact details of all patients who visited that day and calling them yourself
E Arranging for the receptionist to give each patient a one page explanation of the project and asking each patient if they are willing to have their contact details given to you
F don't know

25. You plan to conduct interviews with family members of patients who have attended emergency departments about what mattered most to them.

Which ONE of the following selection criteria is ethically fair? ***Please fill in ONE answer***.

A all of the family members of patients who attended on a selected day
B only partners or parents of patients
C only members of families whose principal language is English
D any family member who volunteers
E don't know

26. You plan to conduct research to assess how motivation affects the maintenance of mobility and flexibility in older patients with arthritis. Weekly gym sessions will be provided to measure mobility.

How would you manage the risks and benefits of this research? ***Please fill in ONE answer.***

A by keeping the gym sessions to the tolerable level of effort for the least mobile patient
B by using an escalating scale of effort for gym sessions as weekly targets
C by advising all patients of a validated scale of mobility that they are expected to reach
D by using only an individual measure of mobility achievement for each patient
E don't know

27. You plan to conduct research on the factors that affect inter-professional relationships among health professionals in a regional multi-disciplinary health service that employs more than 20 staff.

In seeking the consent of all the professionals involved, which ONE of the following undertakings should be given to participants? ***Please fill in ONE answer.***

A	that you will only use pseudonyms and not identify individuals
B	that you will remove information that identifies them and that of any professionals they refer to in an interview
C	that you will retain identifying information about them and any others they refer to, but will not identify anyone in a report of the research
D	that you will disclose identifying information of all interviewees and others referred to in interviews in a confidential report to the clinic staff
E	don't know

28. While conducting a survey about risk factors for depression from patients in a GP waiting room, you notice that a participant has a significantly high score on a depression rating scale.

As a researcher, your responsibility is to: ***Please fill in AS MANY answers as required.***

A	include information on the participant information sheet about symptoms of depression
B	include information on the participant information sheet about where to seek help
C	approach the patient and inquire about symptoms of depression
D	refer the patient to a counselling or mental health service
E	inform practice staff about the patient's self report so they can inform the GP
F	inform the patient's GP about the patient's self report
G	don't know

D. TRAINING AND EDUCATION IN HUMAN RESEARCH ETHICS

Please indicate below the type of information, education or training about human research ethics that would be useful for you.

Thank you for your participation.

By submitting this survey, you consent to participating in this research.

Following the submission of this page, you will no longer have access to this survey.

Thank you for your valuable contributions to this study.

The MD Project team will provide an annual update of this project to all academic staff involved in the MD project Program via email or online link.

Should you have any questions please contact: medicine.mdproject@sydney.edu.au

[i] This survey was based upon previous work by:

Babl FE, Sharwood LN. Research governance: current knowledge among clinical researchers. Medical Journal of Australia. 2008;188(11):649-52.

Weston KM, Mullan JR, Hu W, Thomson C, Rich WC, Knight-Billington P, et al. Academic guidance in medical student research: how well do supervisors and students understand the ethics of human research? Journal of Academic Ethics. 2016;14(2):87-102.
