# Supplementary material for: Supervising Ethically: Applied Research Ethics Knowledge Among Supervisors of Medical Student Research Projects
Source: Med Sci Educ. 2026 Sep 9;36(5):2327–36. doi: 10.1007/s40670-026-02876-4 (PMC13723458; doi:10.1007/s40670-026-02876-4)
Supplement: Supplementary file 2 [file 40670_2026_2876_MOESM2_ESM.pdf]

Supplementary Table S1. Scenario-based ethics performance by supervisor characteristics

STAFF CATEGORY

Scenario-based ethics item	Overall	Academic/affiliate (n=44)	Conjoint medical/health (n=17)	Non-academic (n=3)	p value
Use of medical records: specific consent	53/64 (82.8%)	38/44 (86.4%)	13/17 (76.5%)	2/3 (66.7%)	0.371
Written consent requirements	24/64 (37.5%)	19/44 (43.2%)	5/17 (29.4%)	0/3 (0.0%)	0.306
Participant information statement requirements	40/64 (62.5%)	31/44 (70.5%)	7/17 (41.2%)	2/3 (66.7%)	0.112
Guiding ethical principle (respect)	28/64 (43.8%)	19/44 (43.2%)	6/17 (35.3%)	3/3 (100.0%)	0.154
Clinic-based recruitment strategy	13/64 (20.3%)	12/44 (27.3%)	1/17 (5.9%)	0/3 (0.0%)	0.146
Fair participant selection	24/64 (37.5%)	16/44 (36.4%)	8/17 (47.1%)	0/3 (0.0%)	0.393
Risk management in mobility research	43/64 (67.2%)	30/44 (68.2%)	11/17 (64.7%)	2/3 (66.7%)	1.000
Privacy/confidentiality in interview research	53/64 (82.8%)	34/44 (77.3%)	17/17 (100.0%)	2/3 (66.7%)	**0.031**
Management of incidental findings	17/64 (26.6%)	12/44 (27.3%)	5/17 (29.4%)	0/3 (0.0%)	0.788

MEDICAL DEGREE

Scenario-based ethics item	Overall	Yes (n=36)	No (n=28)	p value
Use of medical records: specific consent	53/64 (82.8%)	31/36 (86.1%)	22/28 (78.6%)	0.513
Written consent requirements	24/64 (37.5%)	16/36 (44.4%)	8/28 (28.6%)	0.193
Participant information statement requirements	40/64 (62.5%)	21/36 (58.3%)	19/28 (67.9%)	0.435
Guiding ethical principle (respect)	28/64 (43.8%)	18/36 (50.0%)	10/28 (35.7%)	0.253
Clinic-based recruitment strategy	13/64 (20.3%)	1/36 (2.8%)	12/28 (42.9%)	**<0.001**
Fair participant selection	24/64 (37.5%)	15/36 (41.7%)	9/28 (32.1%)	0.435
Risk management in mobility research	43/64 (67.2%)	24/36 (66.7%)	19/28 (67.9%)	0.920
Privacy/confidentiality in interview research	53/64 (82.8%)	34/36 (94.4%)	19/28 (67.9%)	**0.007**
Management of incidental findings	17/64 (26.6%)	11/36 (30.6%)	6/28 (21.4%)	0.412

PHD

Scenario-based ethics item	Overall	Yes (n=50)	No (n=14)	p value
Use of medical records: specific consent	53/64 (82.8%)	41/50 (82.0%)	12/14 (85.7%)	1.000
Written consent requirements	24/64 (37.5%)	18/50 (36.0%)	6/14 (42.9%)	0.639
Participant information statement requirements	40/64 (62.5%)	33/50 (66.0%)	7/14 (50.0%)	0.274
Guiding ethical principle (respect)	28/64 (43.8%)	21/50 (42.0%)	7/14 (50.0%)	0.594
Clinic-based recruitment strategy	13/64 (20.3%)	13/50 (26.0%)	0/14 (0.0%)	0.055
Fair participant selection	24/64 (37.5%)	18/50 (36.0%)	6/14 (42.9%)	0.639
Risk management in mobility research	43/64 (67.2%)	32/50 (64.0%)	11/14 (78.6%)	0.356
Privacy/confidentiality in interview research	53/64 (82.8%)	39/50 (78.0%)	14/14 (100.0%)	0.103
Management of incidental findings	17/64 (26.6%)	13/50 (26.0%)	4/14 (28.6%)	1.000

PRIOR ETHICS TRAINING

Scenario-based ethics item	Overall	Yes (n=55)	No (n=9)	p value
Use of medical records: specific consent	53/64 (82.8%)	44/55 (80.0%)	9/9 (100.0%)	0.338
Written consent requirements	24/64 (37.5%)	21/55 (38.2%)	3/9 (33.3%)	1.000
Participant information statement requirements	40/64 (62.5%)	36/55 (65.5%)	4/9 (44.4%)	0.277
Guiding ethical principle (respect)	28/64 (43.8%)	22/55 (40.0%)	6/9 (66.7%)	0.163
Clinic-based recruitment strategy	13/64 (20.3%)	12/55 (21.8%)	1/9 (11.1%)	0.672
Fair participant selection	24/64 (37.5%)	21/55 (38.2%)	3/9 (33.3%)	1.000
Risk management in mobility research	43/64 (67.2%)	40/55 (72.7%)	3/9 (33.3%)	**0.049**
Privacy/confidentiality in interview research	53/64 (82.8%)	46/55 (83.6%)	7/9 (77.8%)	0.646
Management of incidental findings	17/64 (26.6%)	15/55 (27.3%)	2/9 (22.2%)	1.000

HREC REVIEWER EXPERIENCE

Scenario-based ethics item	Overall	Yes (n=19)	No (n=41)	p value
Use of medical records: specific consent	53/64 (82.8%)	14/19 (73.7%)	35/41 (85.4%)	0.301
Written consent requirements	24/64 (37.5%)	9/19 (47.4%)	15/41 (36.6%)	0.428
Participant information statement requirements	40/64 (62.5%)	11/19 (57.9%)	27/41 (65.9%)	0.552
Guiding ethical principle (respect)	28/64 (43.8%)	8/19 (42.1%)	18/41 (43.9%)	0.896
Clinic-based recruitment strategy	13/64 (20.3%)	4/19 (21.1%)	9/41 (22.0%)	1.000
Fair participant selection	24/64 (37.5%)	8/19 (42.1%)	13/41 (31.7%)	0.432
Risk management in mobility research	43/64 (67.2%)	15/19 (78.9%)	25/41 (61.0%)	0.170
Privacy/confidentiality in interview research	53/64 (82.8%)	16/19 (84.2%)	34/41 (82.9%)	1.000
Management of incidental findings	17/64 (26.6%)	6/19 (31.6%)	10/41 (24.4%)	0.558

NOTES

Values are correct responses, n/N (%).

P values are two-sided. Pearson chi-square tests were used where expected cell counts were adequate; Fisher exact tests were used otherwise.

Conjoint medical and conjoint health professional staff were combined for analysis to match the staff-category reporting in the manuscript.

HREC reviewer analyses exclude four participants with missing reviewer-status data (N=60).

Subgroup analyses were exploratory and no adjustment was made for multiple comparisons; nominal p values <0.05 should therefore be interpreted cautiously.
